# Pseudopregnancy in goats: Sonographic prevalence and associated risk factors in Khartoum State, Sudan

**DOI:** 10.14202/vetworld.2018.525-529

**Published:** 2018-04-23

**Authors:** Areeg Mohamed Almubarak, Naglaa Abd Elhakeem Abass, Majdi Elnaim Badawi, Mohamed Tagelddin Ibrahim, Abdelhamid Ahmed Elfadil, Rihab Mohamed Abdelghafar

**Affiliations:** 1 Department of Veterinary Medicine and Animal Surgery, College of Veterinary Medicine, Sudan University of Science and Technology, P.O. Box 204, Hilat Kuku, Khartoum North, Sudan; 2 Department of Preventive Medicine and Public Health, College of Veterinary Medicine, Sudan University of Science and Technology, P.O. Box 204, Hilat Kuku, Khartoum North, Sudan; 3 Department of Animal Production Science and Technology, College of Science and Technology of Animal Production, Sudan University of Science and Technology, P.O. Box 204, Hilat Kuku, Khartoum North, Sudan

**Keywords:** goat, hydrometra, prevalence, risk factors, Sudan, ultrasound

## Abstract

**Aim:**

This study was conducted to estimate the prevalence of pseudopregnancy in goats and to investigate potential risk factors associated with the condition in Khartoum State.

**Materials and Methods:**

A cross-sectional study was carried out from March 2015 to February 2016. A total of 378 female goats which presented to the Veterinary Teaching Hospital, College of Veterinary Medicine, Sudan University of Science and Technology, for routine ultrasonographic pregnancy diagnosis were examined. Ultrasound scanning was performed using a real-time scanner equipped with dual-frequency (3.5-5 MHz) curvilinear transducer.

**Results:**

The results showed that the prevalence of pseudopregnancy in goats in Khartoum State was 10.6%. Risk factors such as general body condition (*χ*^2^=5.974; p=0.05), age (*χ*^2^=11.760; p=0.0129), type of estrus (*χ*^2^=12.794; p=0.000), and previous reproductive performance (*χ*^2^=13.397; p=0.020) showed significant association (p≤0.05) with the occurrence of pseudopregnancy in the univariate analysis. Breed (*χ*^2^=12.627; p=0.082), milk yield (*χ*^2^=5.951; p=0.114), type of feeding (*χ*^2^=1.721; p=0.190), season (*χ*^2^=2.661; p=0.264), locality (*χ*^2^=7.66; p=0.264), parity number (*χ*^2^=0.451; p=0.767), and rearing system (*χ*^2^=1.593; p=0.451) were not significantly associated with pseudopregnancy.

**Conclusion:**

The prevalence of pseudopregnancy in goats in Khartoum State was 10.6%. Pseudopregnancy in goats is significantly associated with age, type of estrus, general body condition, and previous reproductive performance. This study showed for the first time that pseudopregnancy is a real reproductive problem in goats in Khartoum State.

## Introduction

During the last decade, interest in goats has increased worldwide due to their importance in agricultural systems in low-income countries and an increased demand for goat products in developed countries [1]. Sudan possesses over 100 million head of livestock population; among this population, the estimated population of goats is about 31 million head [2]. This population figure puts Sudan as a leading livestock producer in Africa and Arab countries [3].

As reproduction is the backbone of the animal production chain, it is essential to increase the reproduction efficiency in goats [4]. Reproductive ultrasonography in small ruminants is the most efficient diagnostic tool for managing reproduction [5]. It has revolutionized the knowledge of reproductive technology [6]. B-mode ultrasound is used on a large scale to monitor reproductive status in small ruminants. It is considered a simple, non-invasive, rapid, and reliable method for detecting pregnancy, estimating litter size and fetal weight, and determining gestational age [7-9]. It is useful in the diagnosis of pathological conditions that reduce fertility and reproductive performance in females such as pseudopregnancy, pyometra, ovarian cysts, and metritis [5,10].

Pseudopregnancy is an anestrus condition in which fluid accumulates inside the uterus with the persistence of corpus luteum (CL) and absence of fetus and/or placentomes [10-12]. It is an important pathological condition because it causes temporary infertility in dairy goats [13], and it is the most common uterine pathology affecting fertility [5,14]. Hydrometra and mucometra are the terms used as synonymous with pseudopregnancy in goats [15]. The etiology and pathophysiology of the condition are not well clarified [16,17]. It is always associated with high plasma progesterone level secreted by a persistent functional CL, cessation of cyclical activity, and a variable degree of abdominal distension [18]. The condition is incidentally found during routine pregnancy diagnosis of mated animals. However, it also occurs in unmated anestrus does during the breeding and non-breeding seasons [16,19]. Hydrometra in goats has been associated with disturbances in either luteotrophic or luteolytic mechanism during the ovarian cycle. Moreover, prolactin may also be associated with the development of hydrometra in goats [19,20].

In temperate areas, the incidence of hydrometra in goats varies between 3.0% and 20.8% [21]. The incidence of hydrometra increases with milk yield and age of the dam and may accompany fetal death [16,21,22]. Hydrometra in goats has also been predisposed by genetics as reported by Hesselink and Elving [23]. In tropics, reports of pseudopregnancy are scarce [24]. In Sudan, only two cases of hydrometra have been reported in goats [25,26]; however, the prevalence and potential risk factors associated with pseudopregnancy in goats are unknown. Hence, the present study was conducted in Khartoum State of Sudan to determine the prevalence and associated risk factors of pseudopregnancy in goats.

## Materials and Methods

### Ethical approval

The study was approved by the Research Committee of the College of Veterinary Medicine, Sudan University of Science and Technology (SUST).

### Study area

The present study was carried out at the Veterinary Teaching Hospital (VTH), College of Veterinary Medicine, SUST, Hilat Kuku, Khartoum North. The VTH received goats from all localities of Khartoum State for routine sonographic pregnancy diagnosis; therefore, it was selected for the present study.

Khartoum State lies in central Sudan in the semi-arid zone between latitude 15° 32.799´N and longitude 32° 32.0166´E in an area about 28.165 km^2^. The average annual temperature in Khartoum State ranges between 22.7°C and 37.1°C, with a mean annual rainfall of 156.8 mm. Geographically, Khartoum State is divided into seven localities, namely Khartoum, Jebal Aulya, Khartoum Bahri, Sharg El Nile, Um Durman, Karari, and Um Badah. The goat population in Khartoum State is around 651,052 [2].

### Study design, animals, and duration

This cross-sectional study was conducted from March 2015 to February 2016. A total of 378 does of different breeds with ages between 6 months and 14 years were included in the study. All goats were put into general health status categories as poor, moderate, and good. Age was determined according to the date of birth (if known) or by using dentition formula according to Smith and Sherman [1]. Sample size was calculated based on 30.4% prevalence reported by Lopes *et al*. [24] and 95% confidence interval and 5% desired precision according to Thrusfield [27]. A pre-tested questionnaire was developed, and all data related to the study objectives were recorded including breed, age, milk production, rearing system, type of feeding, type of estrus, parity number, general body condition, previous reproductive performance, locality, type of insemination, and season.

### Ultrasound technique

Ultrasound scanning was conducted using B-mode real-time scanner (Aquila Vet, Pie medical Easote, the Netherlands) equipped with a dual-frequency (3.5-5 MHz) transabdominal curvilinear probe. Animals were put in a supine position on a specially designed table. Sufficient amount of ultrasonic gel was applied to the shaved ventral abdomen before scanning as described by Goddard [28]. Sonographic pictures were printed on thermal papers (Sony Corporation, Type 1, Normal, UPP-110S, 1-7-1, Konan, Minato-KU, Tokyo, Japan) using a video graphic printer UP-895EC (Sony Co. Tokyo, Japan).

### Statistical analysis

Statistical analysis was performed using the Statistical Package for the Social Sciences (SPSS) version 22.0 (SPSS Inc., Chicago, USA). Descriptive statistics of the variables were obtained. Univariate analysis using Chi-square test was done to determine whether pseudopregnancy prevalence differed significantly between the levels of selected risk factors. p≤0.05 was considered statistically significant.

## Results

### Prevalence and risk factors

In the present study, 40 out of 378 examined goats in Khartoum State were diagnosed with pseudopregnancy, i.e. 10.6% prevalence proportion. The prevalence of pseudopregnancy increased with age (*χ*^2^=11.760; p=0.0129). Does with induced estrus were highly associated with pseudopregnancy (χ^2^=12.794; p=0.000).

Furthermore, a significant association (*χ*^2^=13.397; p=0.020) was found between the previous history of the dam and pseudopregnancy; 21/221 (9.5%) were diagnosed among goats with no previous reproductive problems, while 8/26 (30.8%) had a previous history of pseudopregnancy.

Our study also showed a significant association (*χ*^2^=5.974; p=0.05) between the prevalence of pseudopregnancy and the general condition of the dam. Out of 292 goats with good body condition, 37 (12.7%) were diagnosed pseudopregnant. Three (3.7%) out of 82 goats of moderately body condition were diagnosed as pseudopregnant. Pseudopregnancy was not diagnosed among goats with the poor body condition.

Results of the present study showed that milk yield (*χ*^2^=5.951; p=0.114), breed of the goat (*χ*^2^=12.627; p=0.082), type of feeding (*χ*^2^=1.721; p=0.190), season (*χ*^2^=2.661; p=0.264), locality (*χ*^2^=7.66; p=0.264), parity number (*χ*^2^=0.451; p=0.767), and rearing system (*χ*^2^=1.593; p=0.451) were not significantly associated with pseudopregnancy (Table-1). One risk factor (type of insemination) was not subjected to statistical analysis because all animals in the present study were naturally mated (constant parameter).

**Table-1:** Univariate analysis of the association of potential risk factors with pseudopregnancy in goats using the Chi-square test.

Factor	Number of examined	Number of positives (%)	df	Χ^2^	p
Season					
Summer	100	8 (8)	2	2.661	0.264
Autumn	136	19 (14)			
Winter	142	13 (9.2)			
Locality					
Karari	116	12 (10.3)	6	7.66	0.264
Um Badah	38	5 (13.2)			
Um Durman	18	2 (11.1)			
Sharg El Nile	123	8 (6.5)			
Khartoum Bahri	37	4 (10.8)			
Khartoum	30	7 (23.3)			
Jebal Aulya	16	2 (12.5)			
Breed					
Saanen	226	34 (15)	7	12.627	0.082
Damascus	64	2 (3.1)			
Nubian	33	1 (3)			
Desert	13	1 (7.7)			
Anglonubian	4	0 (0)			
Toggenburg	4	0 (0)			
Damascus×Saanen	28	2 (7.1)			
American	6	0 (0)			
General body condition					
Poor	4	0 (0%)	2	5.974	0.050*
Good	292	37 (12.7%)			
Moderate	82	3 (3.7%)			
Age (years)					
0.5-2	161	14 (8.7)	4	11.760	0.019*
>2-4	156	14 (9)			
>4-6	39	5 (12.8)			
>6-8	13	4 (30.8)			
-8	9	3 (33.3)			
Type of estrus					
Natural	362	34 (9.4)	1	12.794	0.000*
Induced	16	6 (37.5)			
Milk yield					
0	111	11 (9.9)	3	5.951	0.114
0.5-3	74	4 (5.4)			
> 3-6	104	10 (9.6)			
> 6	89	15 (16.9)			
Parity					
Nulliparous	112	11 (9.8)	2	0.351	0.767
Primiparous	78	7 (9)			
Multiparous	188	22 (11.7)			
Previous history					
Normal	221	21 (9.5)	5	13.397	0.020*
Pseudopregnancy	26	8 (30.8)			
Abortion	30	1 (3.3)			
Dystocia	1	0 (0)			
Stillbirth	1	0 (0)			
No history	99	10 (10.1)			
Rearing system					
Open	3	0 (0)	2	1.593	0.451
Close	365	40 (11)			
Mixed	10	0 (0)			
Type of feeding					
Green fodder	14	0 (0)	1	1.721	0.190
Mixed	364	40 (11)			

* p≤0.05 was considered as statistically significant, df=degree of freedom

## Discussion

Pseudopregnancy is a pathological condition characterized by the accumulation of aseptic fluid in uterine lumen with persistence of a functional CL, a variable degree of abdominal distension, and cessation of cyclical activity [18,19,21]. The pathophysiology of the condition is not well understood [16,17]. Diagnosis of pseudopregnancy can easily be made by ultrasound [19,21]. The sonographic diagnosis of pseudopregnancy is based on recognition of fluid in the uterus in the absence of fetuses and placentomes [10].

To the best of the author’s knowledge, this is the first study to investigate and report the prevalence of pseudopregnancy in goats from Sudan and the African continent. In the present study, the prevalence of pseudopregnancy in Khartoum State was found to be 10.6%. Hesselink and Elving [23] reported a prevalence of 10.4% during four estrus seasons in white Dutch dairy goats in the Netherlands. In Brazil, a higher percentage (12.4%) was reported in Toggenburg and Saanen breeds by Souza *et al*. [13]. In the Netherlands, slightly lower prevalence (9%) was found in Saanen goats by Hesselink [21]. An incidence of 3.26% was reported by Batista *et al*. [29] in Canary Island goats. Nevertheless, in Brazil, a higher prevalence (30.4%) was found in Saanen goats by Lopes *et al*. [24]. The difference among these studies could be due to differences in environmental conditions, management, and breed of the animals.

The results of the present study showed highly significant association (p=0.000) between the type of estrus cycle and pseudopregnancy. Similar findings were reported by Batista *et al*. [29], who observed higher frequency in goats made to ovulate using hormonal treatments. Their study reported that, regardless of the season of the year, treatment of the goats with progesterone favored the development of hydrometra. Our findings are also in line with Wittek *et al*. [16], who found the higher frequency in does which had been mated after estrus synchronization with progesterone and pregnant mare serum gonadotropin. Conflicting results have been obtained by Moraes *et al*. [22], who reported 9% pseudopregnancy in goats that were not subjected to any hormonal treatment for estrus induction.

An increase of pseudopregnancy proportion with age was reported in our study (p=0.019). High frequency in elder goats was reported by Wittek *et al*. [16], Hesselink [21], and Batista *et al*. [29], and this is in agreement with the present findings. Concerning the former pathological history of examined goats, the high prevalence of pseudopregnancy (30.8%) was found in goats with the previous report of pseudopregnancy (p=0.020). Similar findings were obtained by Wittek *et al*. [16], who examined a herd of 2434 goats and reported that pseudopregnant goats showed a high risk of developing hydrometra again. Also, a significant association (p=0.05) was found between the general body condition of the goat and pseudopregnancy.

Our study revealed no significant association (p=0.082) between the breed and pseudopregnancy. These findings are in agreement with Wittek *et al*. [16] who reported that the breed of the goats did not influence the incidence of hydrometra. However, it is particularly interesting that the higher prevalence of pseudopregnancy was found in Saanen goats and their crosses. A previous study identified pseudopregnancy as a major problem of Saanen breed raised in northeast part of Brazil [24]. They found that the problem is exacerbated because of lack of ultrasonographic diagnosis in the farms. Batista *et al*. [29] reported that a genetic predisposition might have a role in the high incidence of hydrometra. Moreover, Hesselink and Elving [23] reported a high frequency of hydrometra in daughters of pseudopregnant does of Saanen herd in the Netherlands.

Milk yield was not associated significantly with pseudopregnancy in the present study (p=0.114). This finding is in line with Wittek *et al*. [16] who reported that milk yield did not influence the incidence of hydrometra. On the contrary, Hesselink [21] and Moraes *et al*. [22] found that the high incidence of pseudopregnancy was associated with increased milk yield.

Based on our data, the season was not a significant risk factor for hydrometra (p=0.264). This agrees with previous research of Hesselink and Taverne [10] and Taverne *et al*. [19] who reported that the condition occurs both outside and during the breeding season. In contrast, Wittek *et al*. [16] recorded higher incidence of hydrometra out of the breeding season. This variation could be due to the difference in climatic factors; the previous studies were from temperate regions where goats tend to breed only seasonally, with the breeding season being from July/August to November/December [30]. However, in the tropical zones, goats tend to breed throughout the year [12].

According to the present study, there was no significant association (p=0.767) between parity number and pseudopregnancy. However, pseudopregnancy was diagnosed with a considerable percentage (27.5%) in nulliparous goats. On the contrary, Batista *et al*. [29] reported 43/1321 (3.26%) pseudopregnancy in goats and all of them had kidded at least once.

According to our observations, feeding type and rearing system do not influence the occurrence of pseudopregnancy. These findings agree with Moraes *et al*. [22] who reported that nutritional management and rearing system do not influence the occurrence of pseudopregnancy. Furthermore, no significant association (p=0.264) was found between the locality and pseudopregnancy. Pseudopregnancy was reported from all localities of Khartoum State. The absence of an association between location and pseudopregnancy could be due to the relative similarity in agro-ecology and management systems between the study locations.

## Conclusion

It can be concluded that the prevalence of pseudopregnancy in goats in Khartoum State was 10.6%. This study also showed that pseudopregnancy in goats significantly associated with age, type of estrus, general body condition, and previous reproductive performance.

## Authors’ Contributions

AMA conducted the experimental work (ultrasound scanning) and drafting of the manuscript. RMA and MEB supervised the experimental work and revised the manuscript. NAA helped in data entering, interpretation of the results, and revision of the manuscript. MTI conducted the statistical analysis. AAE designed this study and involved in the manuscript revision. All authors read and approved the final manuscript.
